# Giant Topological Hall Effect and Colossal Magnetoresistance in Heusler Ferromagnet near Room Temperature

**DOI:** 10.1002/adma.202411240

**Published:** 2024-11-27

**Authors:** Premakumar Yanda, Leila Noohinejad, Ning Mao, Nikolai Peshcherenko, Kazuki Imasato, Abhay K. Srivastava, Yicheng Guan, Bimalesh Giri, Avdhesh Kumar Sharma, Kaustuv Manna, Stuart S. P. Parkin, Yang Zhang, Chandra Shekhar, Claudia Felser

**Affiliations:** ^1^ Max Planck Institute for Chemical Physics of Solids 01187 Dresden Germany; ^2^ Deutsches Elektronen‐Synchrotron DESY Notkestr. 85 22607 Hamburg Germany; ^3^ Global Zero Emission Research Center National Institute of Advanced Industrial Science and Technology (AIST) Tsukuba 305‐8569 Japan; ^4^ Max Planck Institute of Microstructure Physics Weinberg 2 D‐06120 Halle (Saale) Germany; ^5^ Indian Institute of Technology‐Delhi Hauz Khas New Delhi 110016 India; ^6^ Department of Physics and Astronomy University of Tennessee Knoxville TN 37996 USA; ^7^ Min H. Kao Department of Electrical Engineering and Computer Science University of Tennessee Knoxville TN 37996 USA

**Keywords:** colossal magnetoresistance, ferromagnetism, martensite structure, shape memory alloy, topological Hall effect

## Abstract

Colossal magnetoresistance (CMR) is an exotic phenomenon that allows for the efficient magnetic control of electrical resistivity and has attracted significant attention in condensed matter due to its potential for memory and spintronic applications. Heusler alloys are the subject of considerable interest in this context due to the electronic properties that result from the nontrivial band topology. Here, the observation of CMR near room temperature is reported in the shape memory Heusler alloy Ni_2_Mn_1.4_In_0.6_, which is attributed to the combined effects of magnetic field‐induced martensite twin variant reorientation (MFIR) and magnetic field‐induced structural phase transformation (MFIPT). This compound undergoes a structural phase transition from a cubic (austenite‐L2_1_) ferromagnetic (FM) to a monoclinic (martensite) antiferromagnetic (AFM), which leads to an effective increase in the size of the Fermi surface and consequently in CMR. Additionally, it exhibits significant anomalous Hall conductivity in both antiferromagnetic and ferromagnetic phases. Furthermore, it demonstrates a giant topological Hall resistivity (THR) $\rho_{\mathrm{yx}}^{T}$ ≈6 µΩ.cm in the vicinity of martensite transition due to the enhanced spin chirality resulting from the formation of magnetic domains with Bloch‐type domain walls. The findings contribute to the understanding of the magnetotransport of Ni‐Mn‐In Heusler alloys, which are prospective candidates for room‐temperature spintronic applications.

## Graphical abstract

Ferromagnetic shape memory Heusler alloy Ni_2_Mn_1.4_In_0.6_ exhibits colossal magnetoresistance (CMR) and giant topological Hall effect (THE) near room temperature, which is attributed to the enhanced spin chirality emerging from the formation of domains and domain walls, which in turn resulted from the combined effect of magnetic field induced martensite twin variant reorientation and magnetic field induced structural phase transformation.

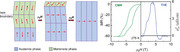

## 1 Introduction

Colossal magnetoresistance (CMR) represents a striking phenomenon observed in certain materials, particularly manganites, where the application of a magnetic field has been shown to dramatically reduce electrical resistance.^[^ ^1^, ^2^, ^3^ ^]^ This property has garnered significant interest due to its potential applications in magnetic sensors, data storage devices, and spintronic technologies. The CMR effect is profoundly intertwined with complex interactions among charge, spin, orbital, and lattice degrees of freedom, giving rise to a multitude of phase transitions and emergent properties.^[^ ^4^, ^5^ ^]^ One of the well‐known materials is hole‐doped rare‐earth manganites, where the double exchange mechanism and Jahn‐Teller distortion are responsible for CMR.^[^ ^6^ ^]^ Recent studies have also indicated that nontrivial band topology plays a crucial role in the manifestation of CMR in various materials.^[^ ^7^, ^8^, ^9^, ^10^ ^]^ Therefore, an understanding of the fundamental mechanisms underlying CMR not only illuminates the emergence of novel electronic phases but also provides a foundation for the design of new materials with tailored electronic and magnetic properties. In this context, Heusler alloys have garnered significant interest due to their diverse electronic properties and great tunability.^[^ ^11^, ^12^, ^13^, ^14^ ^]^ Moreover, they have been identified as topological semimetals, exhibiting topologically protected surface states and anomalous Hall effect.^[^ ^11^, ^15^, ^16^, ^17^, ^18^ ^]^ The presence of such topological features in Heusler alloys broadens their applicability in advanced technologies, particularly in the fields of quantum computing and next‐generation spintronic devices, where the manipulation of spin and charge in topologically non‐trivial states is crucial. Moreover, there is a demand to discover new magnetic topological materials with high magnetic transition temperatures.

Ni‐Mn‐X (X = Ga, In, Sn, and Sb) Heusler alloys, in particular, are distinguished by their exotic magnetoresponsive properties, including magnetic shape memory, magnetic field‐induced strain, magnetocaloric effects, and potential for solid‐state refrigeration and spintronic applications.^[^ ^19^, ^20^, ^21^, ^22^, ^23^ ^]^ These alloys exhibit a complex interplay between structural and magnetic transitions, often characterized by a martensitic transformation that significantly influences their magnetic and electronic behavior.^[^ ^23^, ^24^, ^25^, ^26^ ^]^ Notably, Mn‐rich Ni‐Mn‐In Heusler alloys, such as Ni_2_Mn_1.4_In_0.6_ exhibit a complex phase diagram consisting of intriguing structural and magnetic transitions.^[^ ^21^, ^22^, ^27^, ^28^ ^]^ Despite the extensive knowledge of the structural and magnetic properties of Ni_2_Mn_1.4_In_0.6_, the electronic properties of these materials remain poorly understood. It is therefore imperative to conduct an immediate investigation of magnetotransport studies on high‐quality single crystals, which can help to elucidate the interplay of crystal structure, magnetism, and topology in Mn‐rich Heusler alloys. There have been reports on the Hall effect in both doped and undoped Ni‐Mn‐In Heusler alloys.^[^ ^29^, ^30^, ^31^, ^32^, ^33^, ^34^, ^35^ ^]^ However, the samples were polycrystalline, which restricts the ability to gain a comprehensive understanding of their electronic properties.

In this letter, we synthesize the single crystals of the shape memory Heusler alloy Ni_2_Mn_1.4_In_0.6_ and investigate the magnetotransport properties. The alloy exhibits a FM to AFM transition accompanied by a first‐order structural phase transition from L2_1_‐cubic austenite to a monoclinic martensite structure. Intriguingly, we find negative CMR near room temperature, which is attributed to the combined effect of MFIR and MFIPT. Further, the material shows significant anomalous and/or topological Hall and Nernst effects in both the FM and AFM phases. More importantly, a giant THR in the vicinity of the structural phase transition is demonstrated. The observed topological transport is mainly originating from the formation of magnetic domains with domain walls below the martensite transition. Our results confirm that Ni_2_Mn_1.4_In_0.6_ multitude of intriguing electronic properties, mainly resulting from the interplay of MFIR and MFIPT and the nontrivial band topology of the Fermi surface, which changes across both structures.

## 2 Results and Discussion

### 2.1 Austenite (Cubic) to Martensite (Monoclinic) Structural Phase Transition

**Figure 1:**
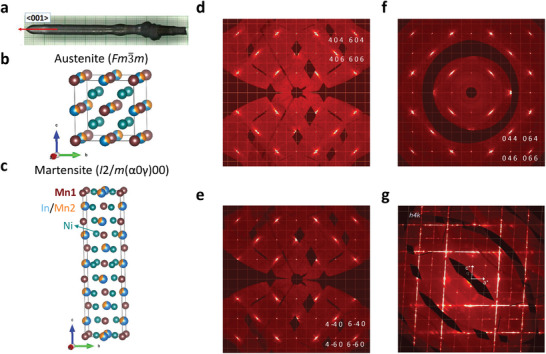
a) Optical image of the crystal grown by optical floating zone (OFZ) of Ni_2_Mn_1.4_In_0.6_. Crystal structure of b) austenite‐L2_1_ and c) martensite phases, visualized by using VESTA software.^[^ ^37^ ^]^ d) (*h 0 l*), e) (*h k 0*), and f) (*0 k l*) sections of the reciprocal space, reconstructed from the SXRD data collected at 360 K. g) (*h 4 l*) section of the reciprocal space, reconstructed from the SXRD data collected at 120 K. Some main reflections are strongly 2D diffused along c* and a*. The q‐vector in gray represents the satellite reflections along the c*‐axis with incommensurate wave vector q = (0, 0, 0.3367(9)), which are located between main reflections. The black areas in the effective reciprocal spaces are due to the beam stop or the gaps between the chip modules in the detector.

**Figure** **1**a shows the Ni_2_Mn_1.4_In_0.6_ crystal, which was grown by the optical floating zone (OFZ) method (Experimental section). The details of phase purity and crystal direction can be found in Supporting Information. The material crystallizes in the cubic‐L2_1_ structure (austenite type) with space group *Fm* $\bar{3}$ *m*. In this structure, Ni atoms are positioned at the Wyckoff site 8c, Mn1 atoms at 4a, and Mn2/In atoms share at 4b, as illustrated in Figure 1b. It is established that Ni‐Mn‐In Heusler alloys undergo a first‐order structural phase transformation from austenite to martensite and result in twinning.^[^ ^21^, ^26^ ^]^ To check this, synchrotron x‐ray diffraction (SXRD) experiments were carried out on a single crystal and the results are shown in Figure 1. As anticipated, Ni_2_Mn_1.4_In_0.6_ also shows martensite transformation to an incommensurate monoclinic structure with modulation vector $q=\left(0,0,\frac{1}{3}\pm \delta \right)$ and possible superspace group *I*2/*m*(α0γ)00 (Figure 1c).^[^ ^21^ ^]^ All Bragg reflections of the high‐temperature phase can be indexed with an *F*‐centered cubic lattice, a = 6.0007 (4) Å. In the second approach, the data were used to produce the sections of reciprocal space. The (*h 0 l*) (*h k 0*) (*0 k l*) sections clearly show the single domain cubic lattice at 360 K (Figure 1d–f). However, 2D diffuse scattering has been observed at every other sharp Bragg reflection. The structural parameters obtained from the analysis of this data by using JANA crystallographic software are shown in Table S1, Supporting Information.^[^ ^36^ ^]^

Upon cooling to 292 K, the crystal undergoes a structural phase transition and the diffraction pattern confirms the lattice distortion to the monoclinic lattice. All Bragg reflections of this phase can be indexed on the body‐centered monoclinic *b*‐unique lattice, a × b × c, together with a modulation wave vector, **q** = (0, 0, 1/3 ± δ), for the satellites employing (3+1)D superspace approach.^[^ ^38^ ^]^ As mentioned earlier, symmetry breaking results in the loss of symmetry from cubic *Fm* $\bar{3}$ *m* to monoclinic *I*2/*m* resulting in the formation of monoclinic twin domains of different orientations. See Nespolo^[^ ^39^ ^]^ for a formal description of the symmetries of twinned crystals. In order to find the nature of the modulation wavevector and diffuse scattering, the crystal cooled down to 120 K. The crystal remained as incommensurately modulated phase and the lattice together with incommensurate wavevector is determined as follows: a = 4.3967(9) Å, b = 5.6017(9) Å, c = 4.3385(9) Å, α = 90°, β = 93.34(2)°, γ = 90°; **q** = (0 0 0.3167(5)) and V = 106.67(3) Å^3^. Further, the coexistence of 2D diffuse scattering, multi‐reticular merohedral twin domains, and incommensurate modulation along the c‐axis makes the diffraction pattern and crystal structure analysis complicated. For simplicity, the reciprocal lattice with only one domain along a*, b*, and c* reciprocal axes of the constructed layers at 120 K has been visualized in Figure S3 (Supporting Information). The major diffuse scattering features near the Bragg reflections observed at 360 K persist down below the martensite phase even when a long‐range order modulated phase is formed (Figure 1g). It appears that not only the diffuse scattering in the austenite phase growing into superlattice peaks below 292 K but also superlattice reflections contribute to diffuse scattering. This indicates short‐range order‐disorder exists as local defects in the sample. Overall, SXRD analysis confirmed the structural phase transition from austenite to an incommensurate martensite structure and the presence of diffuse scattering and martensite twin variant formation.

### 2.2 Metal‐Insulating‐Metal Phase Evolution in Electrical Transport

**Figure 2:**
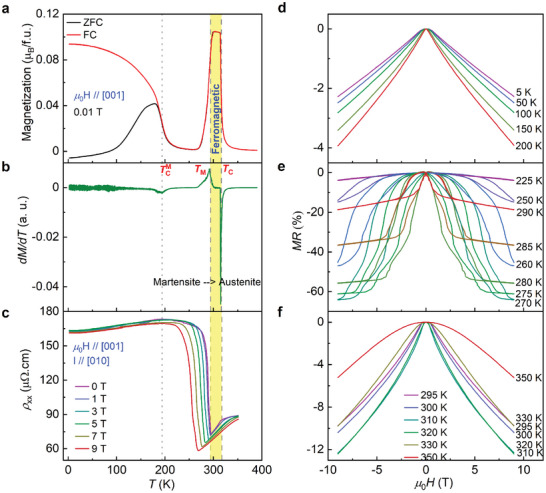
a) Temperature‐dependent magnetization measured under a magnetic field of 0.01 T in ZFC and FC conditions with field applied along [001]. b) First derivative of *T*‐dependent magnetization showing the transitions to ferromagnetic *T*_C_ of austenite, martensite transition (*T*_M_), and Curie temperature of martensite ($T_{C}^{M}$). c) Resistivity as a function of temperature measured at different magnetic fields. The yellow strip indicates the ferromagnetic region. Magnetoresistance in different regions d) 2–200 K (*T* < $T_{C}^{M}$), e) 225–290 K ($T_{C}^{M}$ < *T* < *T*_M_), and f) 295–350 K (*T* > *T*_M_). All these measurements were performed using the configuration with *µ*_0_ *H*//[001] and *I*//[010].

The magnetic measurements, shown in **Figure** **2**a,b, confirmed that Ni_2_Mn_1.4_In_0.6_ exhibits paramagnetic to aFM transition at *T*_C_ = 315 K. The structural phase transition accompanied by FM (high moment) to an AFM (low moment) transition occurs at *T*_M_ ≈ 292 K with the decrease in temperature. Below is the Curie temperature of the martensite $T_{C}^{M}$ ≈ 190 K, the bifurcation between zero‐field cooled (ZFC) and field‐cooled (FC) magnetization can be attributed to the competition between AFM and FM exchange interactions, in agreement with the previous reports.^[^ ^40^, ^41^, ^42^ ^]^ The temperature‐dependent resistivity (Figure 2c) confirms that the material exhibits metallic behavior in both the paramagnetic and FM phases. It shows a kink at *T*_C_, which lends further support to the correlation between magnetism and electronic properties. The resistivity shows a pronounced upturn at the martensite transition, which is indicative of an insulating transition. This can be ascribed to the scattering of charge carriers by magnetic inhomogeneities resulting from twinning. It is noteworthy that, akin to other ferromagnetic Heusler alloys, the resistivity of Ni_2_Mn_1.4_In_0.6_ also violates Mooij's rule.^[^ ^30^, ^31^, ^33^, ^34^ ^]^ According to Mooij's rule,^[^ ^43^ ^]^ the resistivity of an alloy is anticipated to decrease with increasing temperature, provided that the resistivity exceeds 150 µΩ cm^−1^. This is irrespective of the scattering mechanism of the charge carriers. As the temperature is further reduced, the material returns to the metallic state below $T_{C}^{M}$ ≈190 K, indicating the role of competition between AFM and FM interactions.

To further elucidate the relationship between magnetic order and resistivity, we have conducted electrical transport under external magnetic fields in all three regions *T* < $T_{C}^{M}$, $T_{C}^{M}$ < *T* < *T*_M_, and *T* > *T*_M_. Figure 2c shows the temperature‐dependent resistivity at different fields. The structural phase transition shifts to lower temperatures with an increasing magnetic field, indicating the stabilization of the low‐resistance austenite phase and the occurrence of large magnetoresistance. Figure 2d–f illustrates the magnetoresistance at different temperatures, which is calculated using the following formula;

(1) $$MR\;\left(\%\right)=\frac{\rho \left(H\right)-\rho \left(0\right)}{\rho \left(0\right)}\;\times 100\left(\%\right)$$

In all the three regions, negative magnetoresistance is observed, which can be explained based on the *s*‐*d* model where the *s*‐conduction electrons are scattered by localized *d*‐spins. For *T* < $T_{C}^{M}$, it shows negative magnetoresistance of 4% (Figure 2d). Interestingly, it exhibits negative CMR of ≈65% in the vicinity of martensite transition, with a temperature range $T_{C}^{M}$ < *T* < *T*_M_, as shown in Figure 2e. This value of magnetoresistance is higher than that observed in the state‐of‐the‐art Ni‐Mn‐X Heusler alloys.^[^ ^20^, ^44^, ^45^ ^]^ Furthermore, a negative magnetoresistance of less than 13% has been observed for *T* > *T*_M_ (Figure 2f). As previously stated, the resistivity of the martensite phase is observed to increase as a result of scattering from different orientations of the twin boundaries. The application of a magnetic field results in a shift of the *T*_M_ to a lower temperature. In addition, the structural transition to the lower symmetric martensite phase is accompanied by modulation, which can also increase the scattering of conduction electrons. The simultaneous occurrence of all these effects gives rise to an increased negative magnetoresistance.

**Figure 3:**
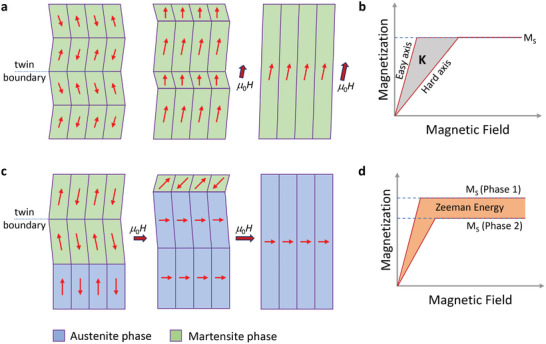
a) Magnetic field‐induced martensite twin reorientation (MFIR). b) Magneto crystalline energy (K) responsible for MFI R. c) Magnetic field‐induced structural phase transformation (MFIPT). d) The difference between the Zeeman energy of austenite and martensite phases responsible for MFIPT.^[^ ^23^ ^]^

It is worth mentioning the characteristics observed in the vicinity of first‐order structural phase transition. The observed MR curves show metamagnetic‐type behavior that is consistent with the isothermal magnetization curves. The main reason for this behavior and CMR in this material can be attributed to magnetic field‐induced strain, as has been reported in ferromagnetic Heusler alloys.^[^ ^19^, ^20^, ^23^, ^24^, ^46^ ^]^ There are two known magnetic field‐induced strain scenarios that can occur in Ni_2_Mn_1.4_In_0.6_ under an applied external magnetic field, as illustrated in **Figure** **3**. The first scenario is MFIR, in which the rearrangement of the microstructure occurs as a result of twin boundary motion triggered by a magnetic field (Figure 3a). If the magnetocrystalline anisotropy energy, K of a martensite twin variant is larger than the energy required for the motion of the twin boundary, K acts as a driving force for MFIR (Figure 3b). Another scenario of MFIPT, as shown in Figure 3c. Here, increasing the magnetic field transforms the crystal structure from martensite to austenite in the coexistence region. The difference in Zeeman energy (Figure 3d) between the two phases acts as the driving force for the MFIPT, provided that the difference is higher than the energy required to move the phase boundaries. The Zeeman energy can be expressed by *E*_Zeeman_  =  µ_0_ *H*Δ*M*, where µ_0_ *H* is magnetic field and Δ*M* is magnetization difference between the transformation phases. Furthermore, this transition leads to an effective increase in electronic Fermi surface size and electrical conductivity (Figure S4, Supporting Information).^[^ ^28^ ^]^ The application of the external magnetic field causes the magnetic moments to reorient along it, making a ground for strong imbalance in different spin polarization populations and strong magnetoresistance.

### 2.3 Anomalous and Topological Hall effects

**Figure 4:**
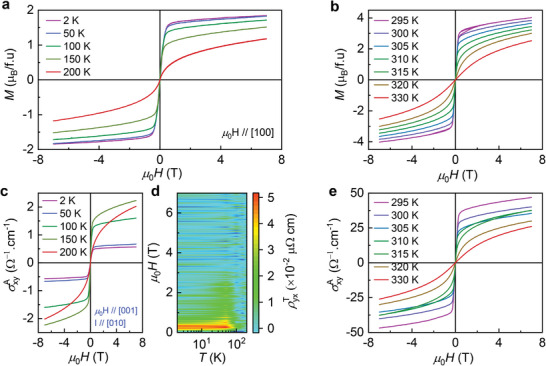
Isothermal magnetization curves measured with magnetic field along [001] in a) *T* < $T_{C}^{M}$ and b) *T* > *T*_M_ regions. c) Anomalous Hall conductivity (AHC) and d) THR in the low‐temperature AFM phase. e) AHC at different temperatures in the FM phase.

**Figure 5:**
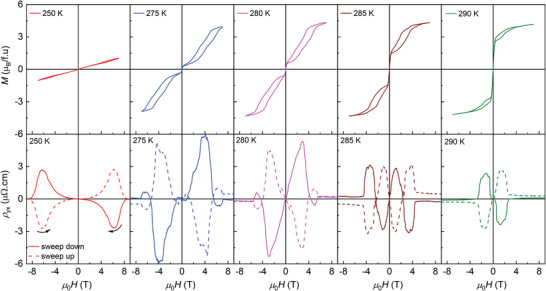
The top and bottom row shows the isothermal magnetization and Hall resistivity curves at fixed temperatures, respectively.

Further, transverse magnetotransport measurements have been performed to unravel the anomalous Hall effect in all three regions, which are presented in **Figures** **4** and **5**. Figure 4a,b shows the isothermal magnetization curves with *µ*_0_ *H*//[001] configuration for *T* < $T_{C}^{M}$ and *T* > *T*_M_, respectively. These measurements confirm that Ni_2_Mn_1.4_In_0.6_ shows magnetization of 1.84 µ_B_ at 2 K and 4 µ_B_ at 295 K. From previous neutron and X‐ray magnetic circular dichroism (XMCD) measurements, it is confirmed that Mn1 atoms order ferromagnetically at room temperature which resulted in higher moment.^[^ ^40^, ^41^, ^42^ ^]^ At low temperatures, Mn2 spins also favor FM alignment, however, Mn1 and Mn2 atoms are connected antiferromagnetically which is mediated by Ni atoms through superexchange interaction.^[^ ^40^ ^]^ Therefore, the observed moment at 2 K is reduced as a result of antiparallel alignment. However, one would expect a higher moment at the Ni site. Interestingly, the possible scenario for this is that the Ni atoms, located between Mn1 and In, are parallel to Mn1. Whereas Ni atoms, located between Mn1 and Mn2, are aligned parallel to Mn2. As a result, the moments of Ni atoms also align antiparallel which gives almost zero moment. Thus, Ni_2_Mn_1.4_In_0.6_ can exhibit exotic properties due to the interplay of intriguing magnetism and topology.

To further investigate this phenomenon, we have carried out Hall measurements in *µ*_0_ *H*//[001] and *I*//[010] configurations. The Hall resistivity can be expressed as ρ_yx_ = ρ^N^ + ρ^A^ + ρ^T^, where ρ^N^, ρ^A^, ρ^T^ are normal, anomalous, and topological Hall resistivities, respectively. The normal Hall resistivity is proportional to ρ^N^ = *R*_0_ *µ*_0_ *H* and anomalous Hall resistivity (AHR) is proportional to ρ^A^ = *b* $\rho_{\mathrm{xx}}^{2}$ *M*, where *R*_0_ and *b* are normal Hall coefficient and proportionality constant, respectively. The magnetization saturates at higher fields which results in zero THR, which gives rise to ρ_yx_ = *R*_0_ *µ*_0_ *H* + *b* $\rho_{\mathrm{xx}}^{2}$ *M*. From this, the plot of ρ_yx_/*µ*_0_ *H* against $\rho_{\mathrm{xx}}^{2}$ *M*/*µ*_0_ *H* represents a straight line and its linear fit produces the slope *b* and intercept *R*_0_. By incorporating these values in the formula ρ^T^ = ρ_yx –_ *R*_0_ *µ*_0_ *H* – *b* $\rho_{\mathrm{xx}}^{2}$ *M*, the THR can be derived. The obtained results are presented in Figure 4c–e. The details of extraction of AHR and THR are provided in Figures S5 and S6, Supporting Information.

The material exhibits anomalous Hall conductivity (AHC), $\sigma_{\mathrm{xy}}^{A}=\frac{\rho_{\mathrm{yx}}}{\rho_{\mathrm{xx}}^{2}+\rho_{\mathrm{yx}}^{2}}$ in the temperature region *T* < $T_{C}^{M}$ which decreases with lowering the temperature, as shown in Figure 4c. The observed AHC at 2 K is $\sigma_{\mathrm{xy}}^{A}$ ≈ 0.56 Ω^−1^ cm^−1^. The presence of ferromagnetic interactions can give rise to a finite Berry curvature which is the origin of the observed AHC. Surprisingly, it also shows the topological Hall effect (THE), as illustrated in Figure 4d. The observed THR is $\rho_{\mathrm{yx}}^{T}$ ≈0.05 µΩ.cm at 2 K. The origin of observed THE can be from real space Berry curvature arising from noncoplanar spin textures below *T* < $T_{C}^{M}$. The noncoplanar spin structures must be the consequence of competition between AFM and FM interactions at low temperatures. On the other hand, we obtained significant AHC in the ferromagnetic phase as well. The obtained AHC is $\sigma_{\mathrm{xy}}^{A}$ ≈ 47 Ω cm^−1^ at 295 K. The presence of significant AHC at room temperature makes this compound promising for practical applications besides magnetic shape memory alloy and magnetic refrigeration technologies.

The transverse resistivity behavior in connection with magnetization in the intermediate region $T_{C}^{M}$ < *T* < *T*_M_ is depicted in Figure 5. The top row of Figures 5 and S7 (Supporting Information) shows the magnetization at different temperatures evidencing the unusual behavior arising due to MFIR and MFIPT to the external magnetic field. The coexistence of MFIR and MFIPT in the vicinity of martensite structural phase transition and their interplay with magnetization can produce complex spin structures. We also measured the Hall resistivity behavior in the same temperature region, as can be seen in the bottom row of Figure 5. The transverse Hall voltage exhibits unusual behavior in the intermediate region. Given that the twin variants respond differently to the field direction, the Hall resistivity curves have been symmetrized separately for the field sweeping both up and down. Interestingly, they show the opposite behavior. The reproducible nature of these curves clearly represents the presence of AHE and THE. The obtained THR is $\rho_{\mathrm{yx}}^{T}$ ∼ 6 µΩ.cm at 275 K, which is higher than the state‐of‐the‐art materials. The magnetic field reorientation of martensitic twin variants might give rise to topological spin textures such as skyrmions due to magnetic inhomogeneities, resulting in enhancement of spin chirality and thus giant THE. In accordance with the AHE, this material shows a significant anomalous and topological Nernst (>1 µV K^−1^) effect as well, as shown in Figure S8 (Supporting Information).

**Figure 6:**
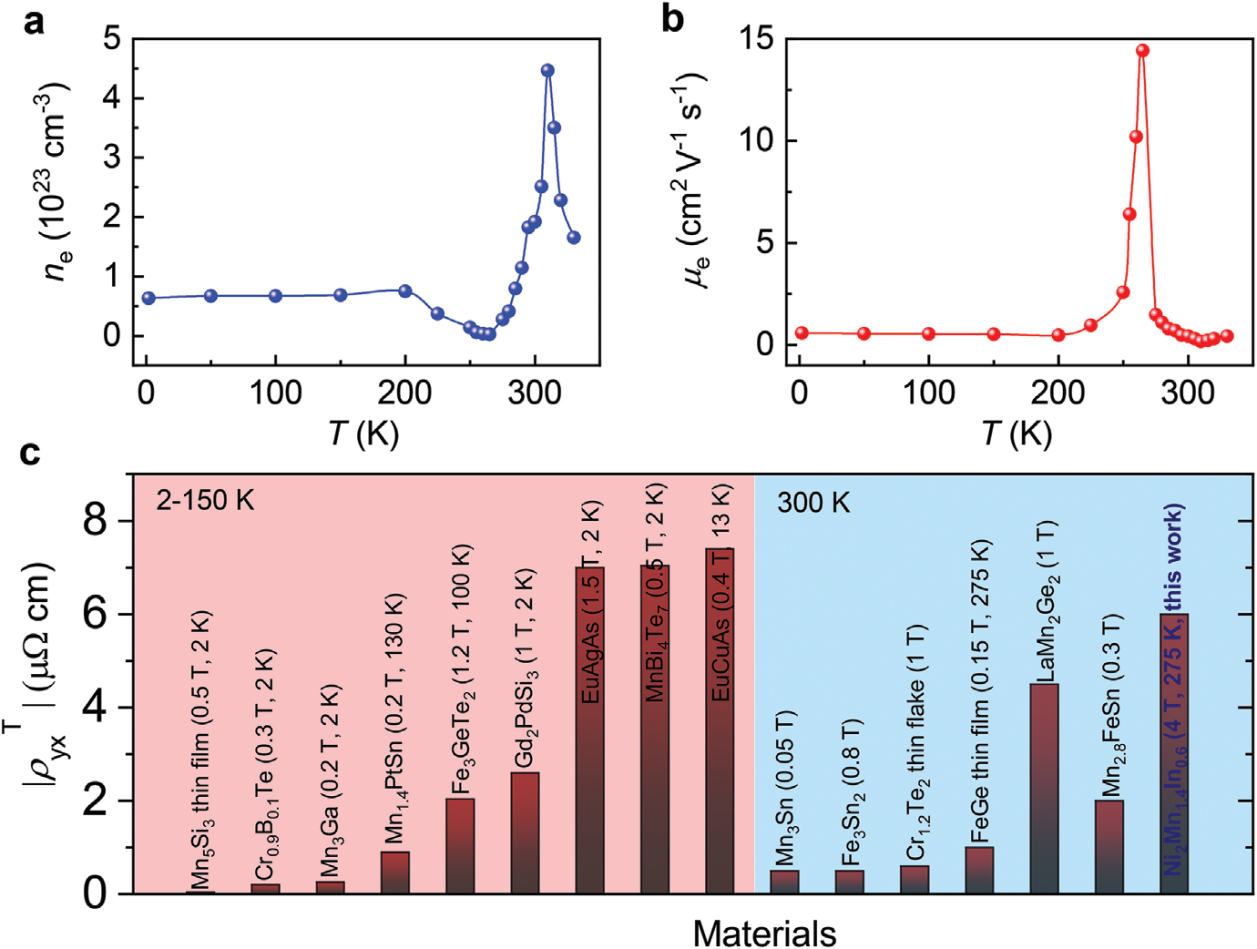
Temperature‐dependent a) carrier concentration obtained from the slope of normal Hall resistivity and b) corresponding carrier mobility. c) Comparison of topological Hall resistivity of Ni_2_Mn_1.4_In_0.6_ with the state‐of‐the‐art materials.^[^ ^47^, ^48^, ^49^, ^50^, ^51^, ^52^, ^53^, ^54^, ^55^, ^56^, ^57^, ^58^, ^59^ ^]^

Furthermore, the material shows dominant electron‐type charge carriers with a carrier concentration (*R*_H_ = 1/*ne*) of ∼10^23^ cm^−3^ extracted from the slope of normal Hall resistivity, as shown in **Figure** **6**a. The carrier density *n*_e_ (6.3  ×  10^22^ cm^−3^ at 2 K and 1.9  ×  10^22^ cm^−3^ at 300 K) increases slightly with temperature and shows an abrupt change around *T*_M_ due to structural phase transition. The corresponding mobilities (Figure 6b) are *µ*_e_ ∼ 0.6 cm^2^ V^−1^ s^−1^ at 2 K and 0.43 cm^2^ V^−1^ s^−1^ at 300 K. The mobility also shows a peak around martensite transition. We further compared the observed THR with the state‐of‐the‐materials (Figure 6c) and our compound outperformed all the materials with $\rho_{\mathrm{yx}}^{T}$ ∼ 6 µΩ.cm at 275 K. This indicates that Ni‐Mn‐In Heusler alloys are promising candidates for not only magnetocaloric but also room‐temperature electronic applications.

Generally, THE occurs in materials with noncoplanar spin textures or nontrivial band topology. Therefore, it is important to understand the band structure and spin textures of the Heusler alloy Ni_2_Mn_1.4_In_0.6_. DFT calculations were employed in order to understand the correlation between the band topology with anomalous and topological transport. In the absence of knowledge of the magnetic structure of this material, we have imaged magnetic domains by using polar magnetic optical Kerr effect (MOKE) microscopy measurements on single crystals. These details will be discussed in the following sections.

### 2.4 Evolution of Electronic Structure Across Transition

For Ni_2_Mn_1.4_In_0.6_, the point group is *m* $\bar{3}$ *m* (O_h_). According to crystal field theory, for such a highly symmetric point group, the $d_{z^{2}}$ and $d_{x^{2}-y^{2}}$ Orbitals form the doubly degenerate e_g_ orbitals, while the *d_xz_*, *d_yz_*, and *d_xy_* orbitals form the triply degenerate t_2g_ orbitals. The four electrons of Mn^3+^, first occupy the triply degenerate t_2g_ orbitals, followed by one electron occupying one of the e_g_ orbitals, resulting in a magnetic moment of 4 µ_B_, which is consistent with the experimental measurement at 295 K. Here, we consider the spin model on the Mn‐based lattice in an external magnetic field defined by the following Hamiltonian:

(2) $$H=J_{1}\sum_{\left\langle i,\;j\right\rangle }S_{i}\cdot S_{j}$$

where ⟨i, j⟩ denote the summation over the nearest exchange couplings and *J*_1_ is the Heisenberg exchange. A large exchange parameter of 131 meV is obtained by DFT calculations. As the temperature decreases, the material undergoes a phase transition, lowering the point group symmetry from *m* $\bar{3}$ *m* (O_h_) to 2/*m* (C_2h_). According to crystal field theory, none of the five *d*‐orbitals remain degenerate in this lower symmetry. Due to the splitting of the e_g_ orbitals, the energy of the previously occupied e_g_ orbital decreases, causing the electron to move back to the t_2g_ orbital. Thus, two singly occupied t_2g_ orbitals contribute to a magnetic moment of 2 µ_B_. Moreover, the distance between the two Mn atoms along the z‐axis decreases from 3 to 2.8 Å, leading to enhanced orbital overlap and increased electron‐electron repulsion. In this scenario, the system will exhibit antiferromagnetic exchange interactions, as the antiparallel spin arrangement minimizes Coulomb repulsion between electrons. The DFT‐calculated exchange energy of *J*_1_ = ‐10.34 meV is also in agreement with this observation.

Further, we performed band structure and AHC calculations for different phases of Ni_2_Mn_1.4_In_0.6_. As depicted in Figure S4 of Supporting Information, the high‐temperature FM phase exhibits a relatively large Fermi surface, which correlates with a significant AHC of ≈47 Ω^−1^ cm^−1^, matching the experimental results. For the monoclinic martensite structure, the space group is *C*2/*m* (No. 12), characterized by symmetries that include 2‐fold rotational symmetry along the y‐axis (C_2y_), mirror symmetry perpendicular to the y‐axis (M_y_), and inversion symmetry (P). In the absence of magnetism, the combined PT symmetry (P for inversion symmetry, T for time‐reversal symmetry) results in a zero Berry curvature across the entire 3D Brillouin zone. However, the emergence of an anti‐parallel spin configuration between two Mn atoms breaks the PT symmetry, leading to a non‐zero Berry curvature. This, in turn, produces AHC of ≈0.5 Ω^−1^ cm^−1^, closely aligning with experimental observations. However, this value is considerably smaller than that of the FM phase, predominantly attributed to the differences in the Fermi surface.

### 2.5 Magnetic Domains and Domain Wall Formation

**Figure 7:**
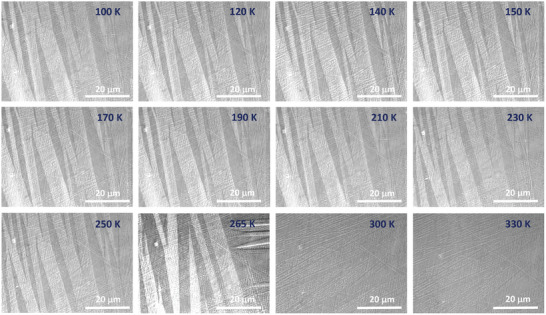
Polar magnetic optical Kerr microscopy images of Ni_2_Mn_1.4_In_0.6_ collected at different temperatures across the austenite‐martensite structural phase transition, showing the formation of domains with Bloch‐type domain walls below the martensite transition. The dark and light areas of magnetic contrast below *T*_M_ correspond to up‐and‐down magnetization.

The polar MOKE microscopy setup is sensitive to the out‐of‐plane component of the magnetization, and the dark and light regions correspond to the up and down magnetization, respectively. The sample is cooled at zero magnetic field and the evolution of the domains with temperature is shown in **Figure** **7**. Interestingly, stripe‐like magnetic domains are observed when the temperature crosses the martensite transition. These magnetic domains persist and become wider as the temperature is lowered to $T_{C}^{M}$. Below this, these domains appear to be unchanged at least down to 100 K. When the sample is heated, these stripe domains disappear at the same temperature at which they appear. No domain formation is observed when the sample is further heated to 340 K. Considering the crystal size, the domain walls could be of the Bloch type.

It is possible that the presence of noncollinear spin texture in these domains or domain walls, which in turn becomes noncoplanar under magnetic field, is responsible for THE below martensite transition and also for CMR. As confirmed by the SXRD measurements, the presence of twin variants and incommensurate modulation may possibly lead to atomic displacements and contribute to the emergence of noncollinear spin structures. The huge change in the formation of these magnetic domains in the vicinity of martensite transition and their response to the magnetic field is responsible for the observed giant THE. Below $T_{C}^{M}$, the domains are almost constant, contributing to the low value of THE. It is interesting to find the chiral spin structure or skyrmions or magnetic bubbles in this interesting material with a centrosymmetric lattice. Additional experiments such as Lorentz transmission microscopy and neutron scattering are required to understand this further.

## 3 Conclusion

We have grown high‐quality single crystals and systematically investigated the electronic properties of shape memory Heusler alloy Ni_2_Mn_1.4_In_0.6_. The material exhibits colossal negative magnetoresistance near room temperature, originating from the combined effect of magnetic field‐induced martensite twin variant reorientation and magnetic field‐induced structural phase transformations under an external magnetic field. Besides, it evidenced significant anomalous and topological Hall and Nernst effects in three regions with distinct magnetic properties. Moreover, a giant topological Hall resistivity, $\rho_{\mathrm{yx}}^{T}$ ≈ 6 µΩ cm^−1^ has been demonstrated close to room temperature, which is higher than state‐of‐the‐art materials. The possible presence of noncoplanar spin textures in the domains or domain walls is responsible for the giant THE, highlighting the strong coupling between magnetism and topology in this material. Our detailed investigation of electronic properties has resolved the scarcity and helps in understanding the magnetotransport in Ni‐Mn‐In Heusler alloys and other related materials. Further, our findings suggest the potential of Heusler alloys for practical applications at room temperature.

## 4 Experimental Section

### Single Crystal Growth and Characterizations

A high‐quality single crystal of Heusler Ni_2_Mn_1.4_In_0.6_ was grown by using the 4‐mirror OFZ method. First, polycrystalline buttons of Ni_2_Mn_1.4_In_0.6 were_ prepared by induction melting of stoichiometric amounts of high‐grade Ni metal pieces, Mn metal (cleaned prior to use), and In wire. Further, these buttons were melted in an induction furnace‐based rod casting instrument to obtain the feed and seed rods for the OFZ method. The single crystal was grown in an Ar atmosphere with a 2 mm hr^−1^ growth rate while seed and feed rods rotated in opposite directions with a speed of 10 mm hr^−1^. The purity and composition of the crystal were confirmed by powder X‐ray diffraction and energy‐dispersive X‐ray spectroscopy, respectively. The Laue diffraction facility was used for orienting the direction of the crystal for the measurements.

Temperature‐dependent synchrotron x‐ray diffraction of the crystal of Ni_2_Mn_1.4_In_0.6_ was measured at beamline P24 of the synchrotron PETRA‐III at DESY, Hamburg, Germany. A Huber 4‐circle diffractometer was used with Euler geometry and equipped with a photon‐counting, LAMBDA‐7.5M CdTe detector. Monochromatic radiation was used at wavelength λ = 0.5 Å. The X‐ray beam had a size of 0.4 × 0.4 mm^2^, and it completely enveloped the crystal. The crystal was heated or cooled within the range of 120–360 K with nitrogen gas, by a CRYOCOOL open‐flow cryostat. Diffraction data were collected by the rotation method. In order to better account for strong and weak reflections, the fine‐slicing method was employed, collecting 3600 frames of Δϕ = 0.1° rotation and 0.5 s exposure time. Then the images binned to 360 frames with 5 s exposure time per degree of phi rotation. X‐ray diffraction data were collected at temperatures of 360 K (cubic phase), and 120 K (monoclinic modulated phase). Indexing, integration, and constructed layers of reciprocal lattice were done with the software CrysAlis Pro.^[^ ^60^ ^]^ The crystal structures were visualized using VESTA crystallographic software.^[^ ^37^ ^]^

### Magnetic and Transport Measurements

Magnetic measurements were carried out using a Superconducting Quantum Interference Device (SQUID), MPMS, and Quantum Design. Electric transport measurements were performed using a 9T‐Physical Property Measurement System (PPMS), Quantum Design. Resistivity was measured using the standard 4 probe method and Hall resistivity was obtained using Hall bar configuration. To measure the Nernst thermopower, a rod‐shaped sample was attached to an alumina plate (heat sink) and a strain gauge heater to create a temperature gradient. Chromel‐constantan thermocouples were used to measure the temperature and voltage differences.

### Theoretical Calculations

First‐principles calculations using the Vienna Ab initio Simulation Package (VASP) were performed within the framework of the generalized gradient approximation (GGA) and Perdew‐Burke‐Ernzerhof (PBE) functionals.^[^ ^61^ ^]^ To better simulate the electronic properties of Heusler alloy Ni_2_Mn_1.4_In_0.6_, the virtual crystal approximation (VCA) in the framework of VASP was employed. For the d orbitals, calculations are conducted using the GGA+U scheme with a U value of 3 eV. Maximally localized Wannier functions (MLWFs) using the WANNIER90 code were constructed,^[^ ^62^ ^]^ and calculate the anomalous Hall conductivities via WannierTools.^[^ ^63^ ^]^ For the AFM phase, the relaxed lattice constants are 4.39, 5.62, and 4.33 Å, with angles of 90°, 93°, and 90°. For the cubic FM phase, the relaxed lattice constant is 6.01 Å, and the angle is 90°.

### Magnetic Optical Kerr Microscopy

Polar Magnetic optical Kerr (MOKE) microscopy is used to image the magnetic domains while the sample is mounted in a CryoVac cryostat using liquid nitrogen as the refrigerant. A Nikon CFI S Plan Fluor ELWD 60XC object lens with a numerical aperture of 0.7 was used.

## Conflict of Interest

The authors declare no conflict of interest.

## Supporting information



Supporting Information

## Data Availability

The data that support the findings of this study are available from the corresponding author upon reasonable request.
