# Supplementary material for: Giant Topological Hall Effect and Colossal Magnetoresistance in Heusler Ferromagnet near Room Temperature
Source: Adv Mater. 2024 Nov 27;37(3):2411240. doi: 10.1002/adma.202411240 (PMC11756049; doi:10.1002/adma.202411240)
Supplement: Supplementary file 1 — Supporting Information [file ADMA-37-2411240-s001.docx]

Supporting Information (SI)

**Giant Topological Hall Effect and Colossal Magnetoresistance in Heusler Ferromagnet Near Room Temperature**

*Premakumar Yanda^1^,* Leila Noohinejad^2^, Ning Mao^1^, Nikolai Peshcherenko^1^, Kazuki Imasato^1,3^, Abhay K. Srivastava^4^, Yicheng Guan^4^, Bimalesh Giri^4^, Avdhesh Kumar Sharma^1^, Kaustuv Manna^2^, Stuart S. P. Parkin^4^, Yang Zhang^6,7^, Chandra Shekhar^1^, & Claudia Felser^1^**

*^1^Max Planck Institute for Chemical Physics of Solids, 01187 Dresden, Germany*

*^2^Deutsches Elektronen-Synchrotron DESY, Notkestr. 85, 22607 Hamburg, Germany*

*^3^Global Zero Emission Research Center, National Institute of Advanced Industrial Science and Technology (AIST), Tsukuba, 305-8569, Japan*

*^4^Max Planck Institute of Microstructure Physics, Weinberg 2, D-06120 Halle (Saale), Germany*

*^5^Indian Institute of Technology-Delhi, Hauz Khas, New Delhi, 110 016, India*

*^6^Department of Physics and Astronomy, University of Tennessee, Knoxville, TN 37996, USA*

*^7^Min H. Kao Department of Electrical Engineering and Computer Science, University of Tennessee, Knoxville, Tennessee 37996, USA*

*E-mail: Premakumar.Yanda@cpfs.mpg.de; Claudia.Felser@cpfs.mpg.de*


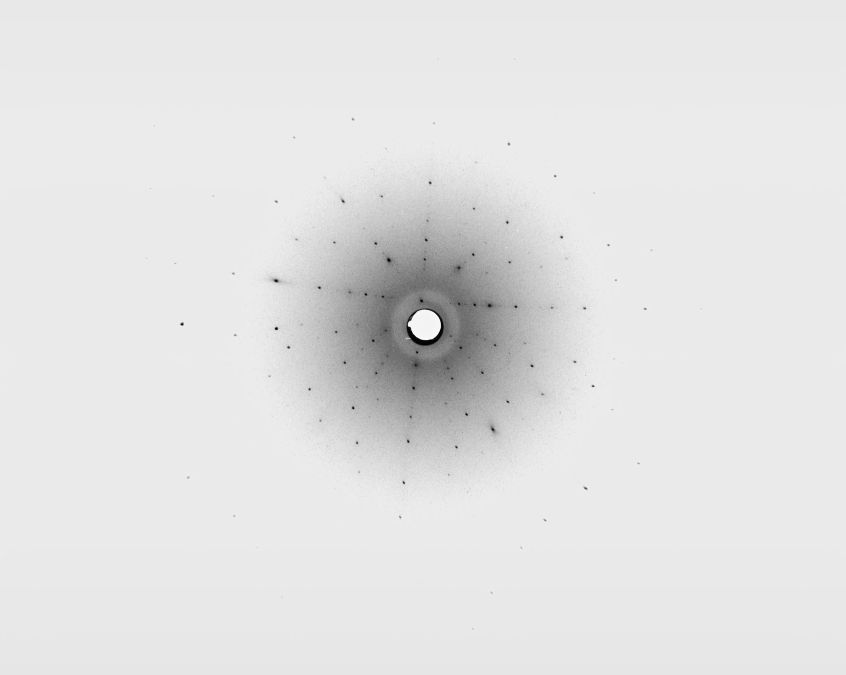


**Figure S1** **Laue x-ray diffraction** showing the (001) direction of single crystal of Ni_2_Mn_1.4_In_0.6_.


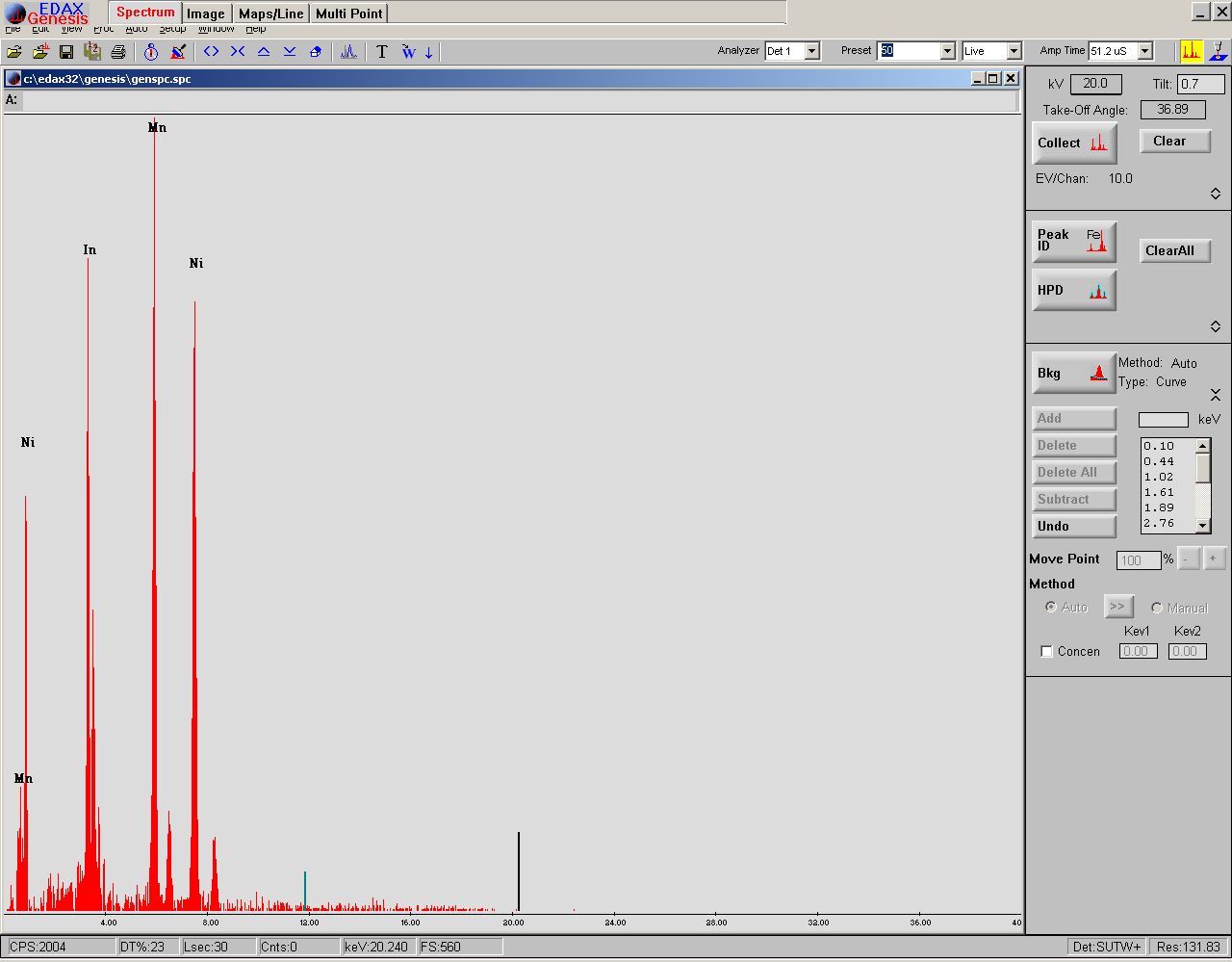

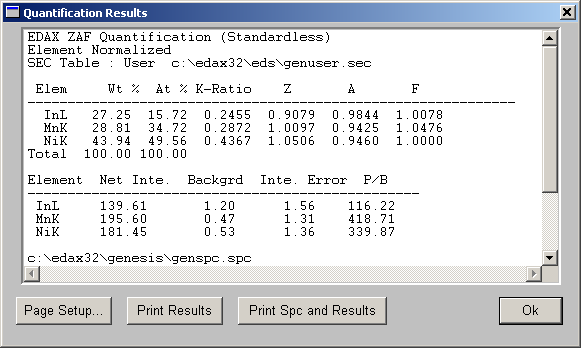


**Figure S2** **Energy dispersive x-ray (EDX) spectrum** results confirming the composition of Ni_2_Mn_1.4_In_0.6_.

**TABLE S1. Atomic coordinates x, y, z, and isotropic atomic displacements (U_iso_) of the structure at 360 K.**

| Atom | Wyckoff  position | *x* | *y* | *z* | U_iso_ (Å^2^) | Occupancy |
| --- | --- | --- | --- | --- | --- | --- |
| Ni | 8c | 0.25 | 0.25 | 0.25 | 0.020(1) | 1 |
| Mn1 | 4b | 0.50 | 0.50 | 0.50 | 0.014(1) | 1 |
| Mn2 | 4a | 0.00 | 0.00 | 0.00 | 0.016(1) | 0.38 |
| In | 4a | 0.00 | 0.00 | 0.00 | 0.016(1) | 0.72 |

Structure solution and refinements have been performed with Jana2006. The cubic structure provides a good fit to the diffraction data measured at 360 K. Space group: *Fm*$\bar{3}$*m*; Cell parameters: *a* = *b* = *c* = 6.0007 (4) Å, *α* = *β* = *γ* = 90 ͦ, *V* = 216.08 (1) Å^3^; GoF = 6.14, Reliability factors: $R$(%) = 4.20, *w*$R$ (%) = 6.03.


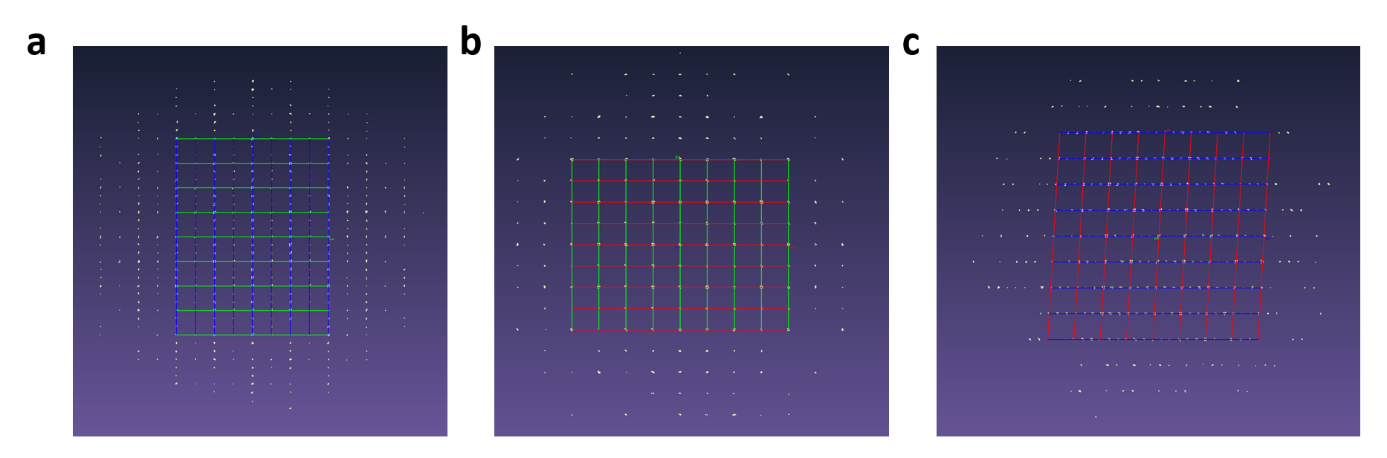


**Figure S3** **Synchrotron x-ray diffraction of martensite phase.** **(a)** a*, bc plane, **(b)** b*, ac plane, and **(c)** c*, ab plane of the reciprocal lattice of crystal at 120 K constructed by using software CrysAlisPro. The grid represents main reflections which are indexed with a I-centered monoclinic b unique lattice and first order satellite reflections are represented with q vector of (0 0 0.3367(9)) along c*. Multi twin domain reflections are neglected for more clarification of single domain lattice.

**Figure S4** **Evolution of electronic band structure and anomalous Hall effects in ferromagnetic (FM) and antiferromagnetic (AFM) phases of Ni_2_Mn_1.4_In_0.6_.** Band structures in **a** high-temperature FM and **c** low-temperature AFM regions. Anomalous Hall conductivity in **c** high-temperature FM and **d** low-temperature AFM regions.


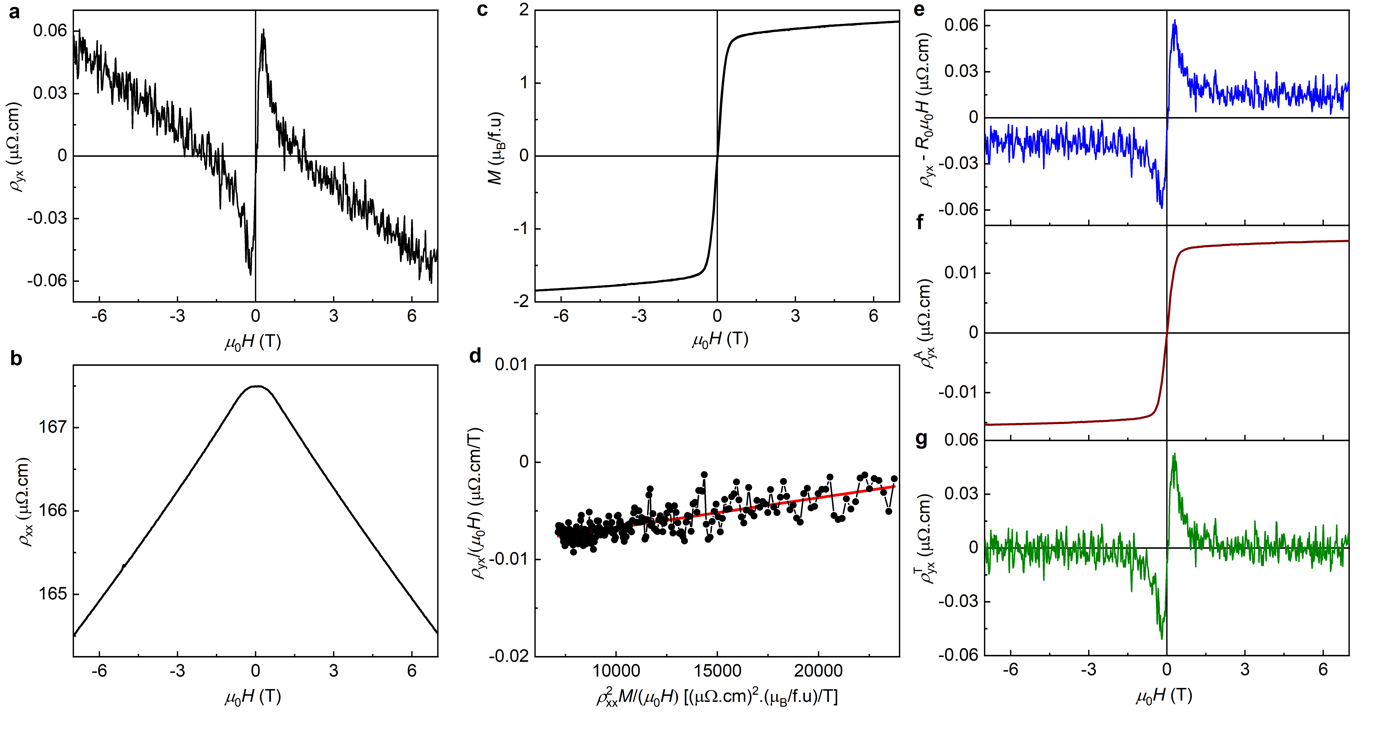


**Figure S5** **Method of extraction of topological Hall resistivity at 2 K. a** Hall resistivity **b** Magnetoresistance **c** Isothermal magnetization **d** ${}_{\mathrm{yx}}$/*μ*_0_*H* against ${}_{\mathrm{xx}}^{2}$*M*/*μ*_0_*H* **e** Hall resistivity without normal Hall **f** anomalous and **g** topological Hall resistivities.


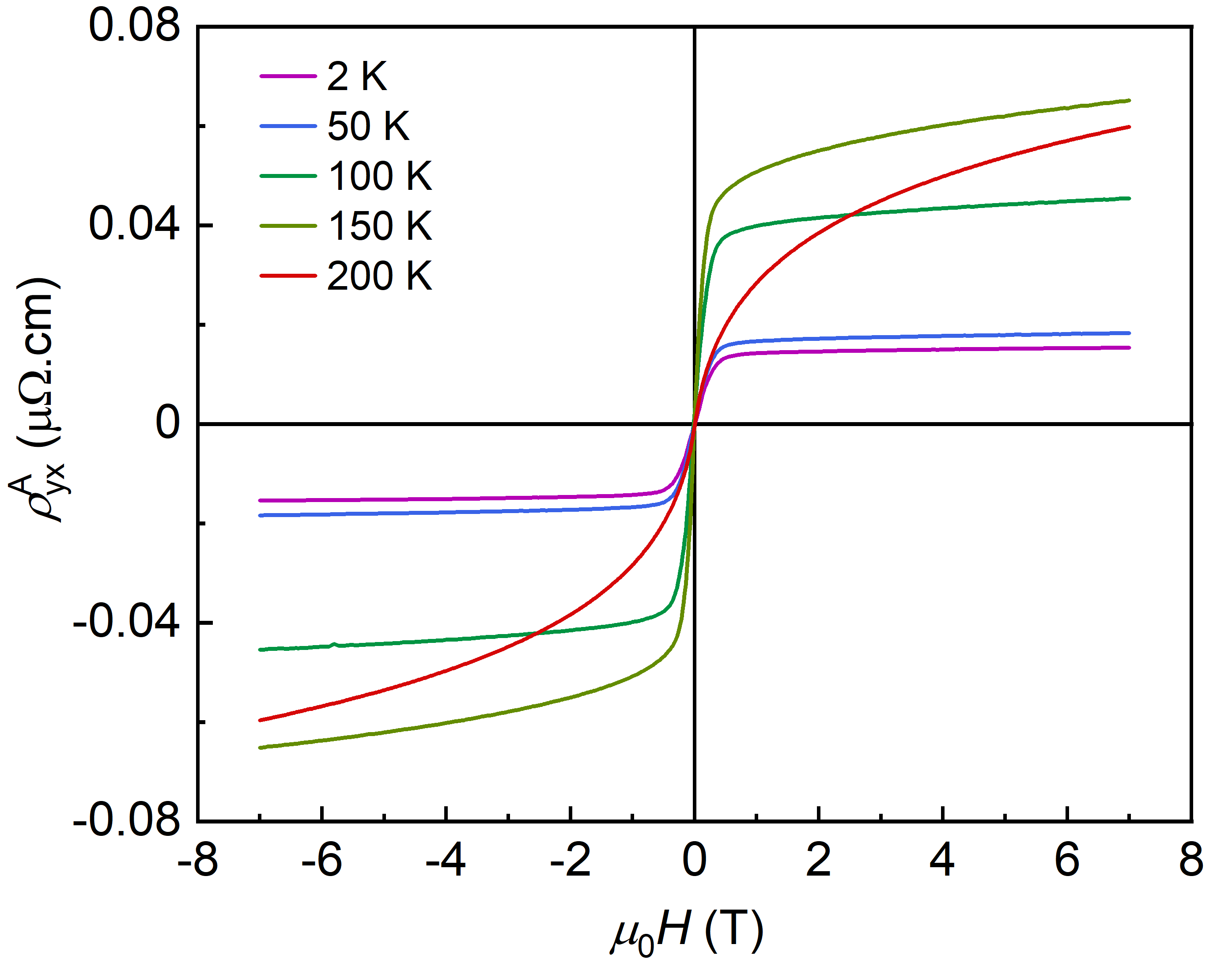


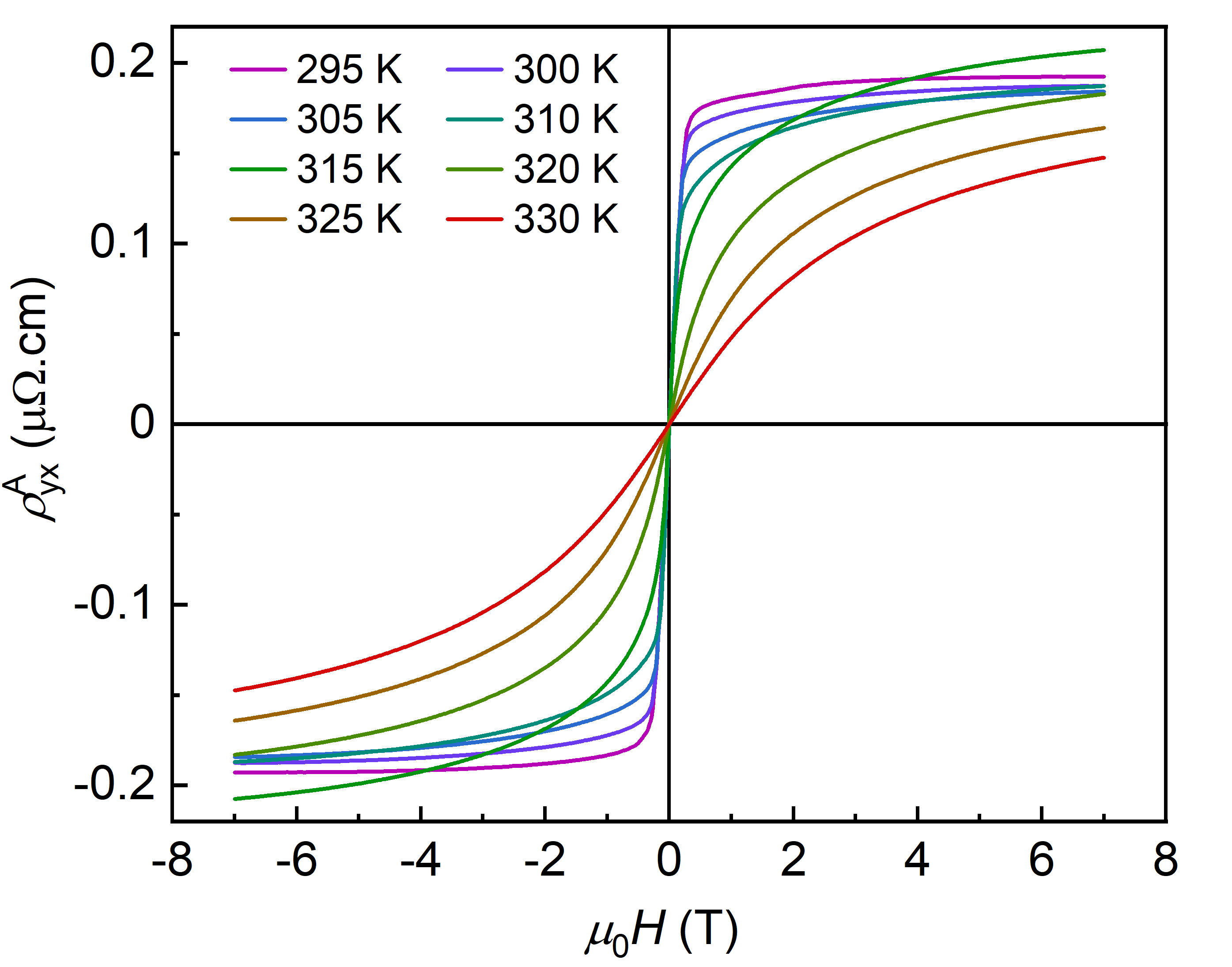


**Figure S6** **Anomalous Hall resistivity in AFM and FM regions.**


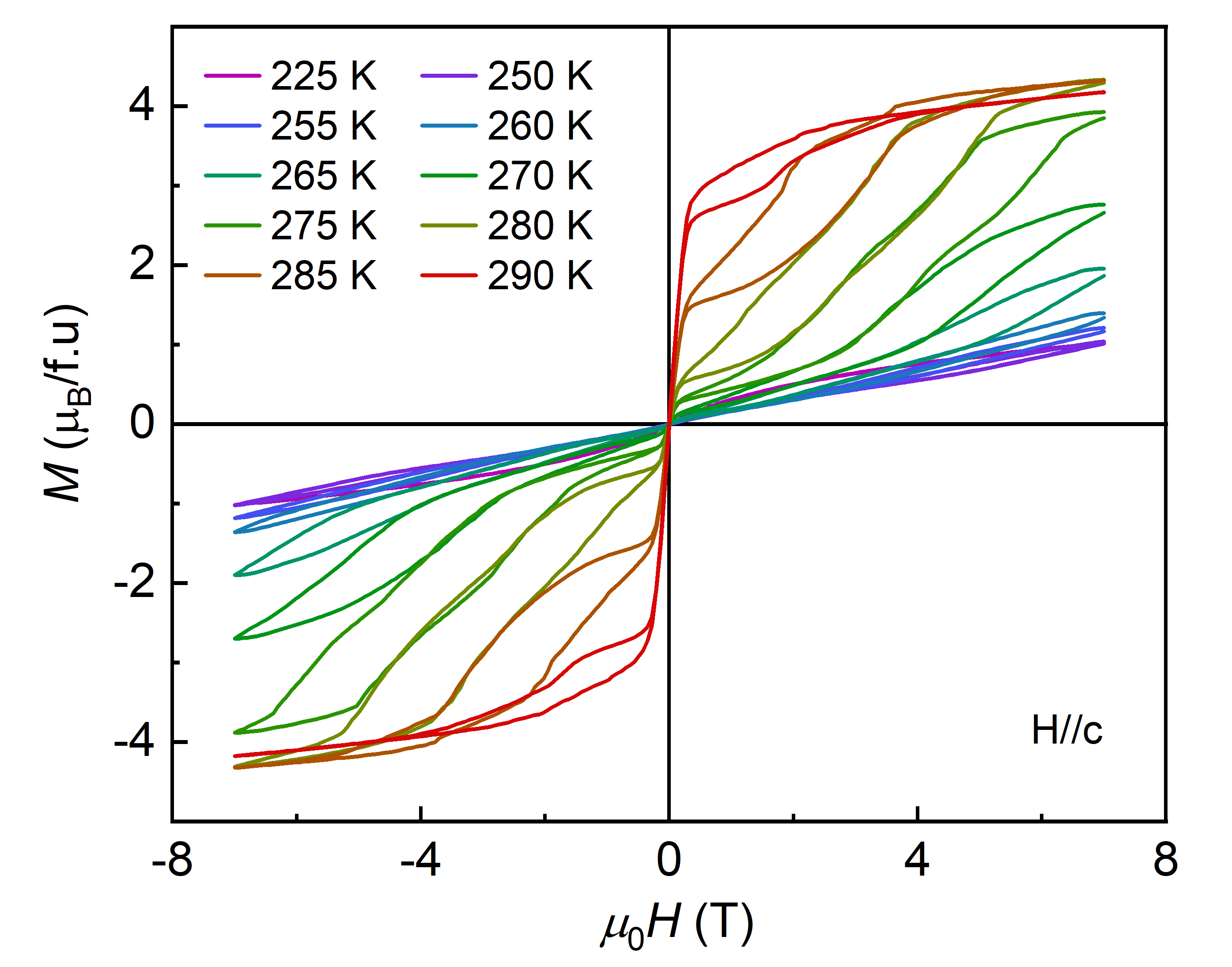


**Figure S7** **Isothermal magnetization curves in the vicinity of martensite structural phase transition.**

**Nernst measurements:**

By applying temperature gradient, field dependent Nernst thermopower of Ni_2_Mn_1.4_In_0.6_ in various temperature regions, *T* < $T_{C}^{M}$ and $T_{C}^{M}$ < *T* < *T*_M_ was measured (Figure S8). While Nernst voltage is small (<0.1 μV/K) when the temperature is near the structural phase transition temperature *T*_M_ ~ 292 K, a larger Nernst signal (>1 μV/K) was observed as a peak within the temperature range of $T_{C}^{M}$ < *T* < *T*_M_. The peak of Nernst signal depending on magnetic field shifts towards higher magnetic field as the temperature decreases, which is consistent with the Hall measurements. As mentioned in main text, the increased electron scattering due to structural phase transition and domain wall formation is the main reason for the observed anomalous and topological Nernst effect as well. And when the temperature is lower than $T_{C}^{M}$, the signal becomes much smaller again compared to the temperature window of 250-285 K.


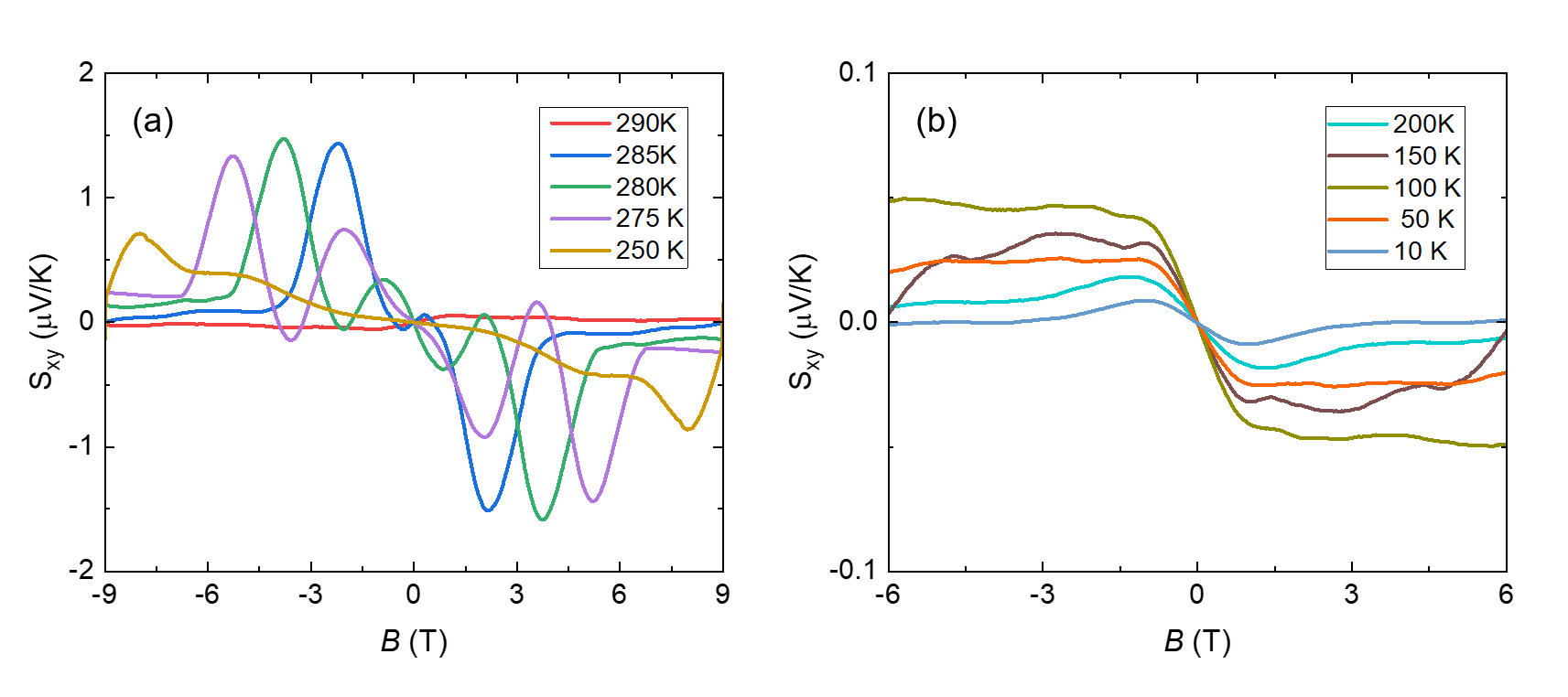


**Figure S8** **Magnetic field dependent Nernst thermopower (S_xy_)** of Ni_2_Mn_1.4_In_0.6_ measured at the temperature range of **(a)** $T_{C}^{M}$ < *T* < *T*_M_ and **(b)** *T* < $T_{C}^{M}$ regions.


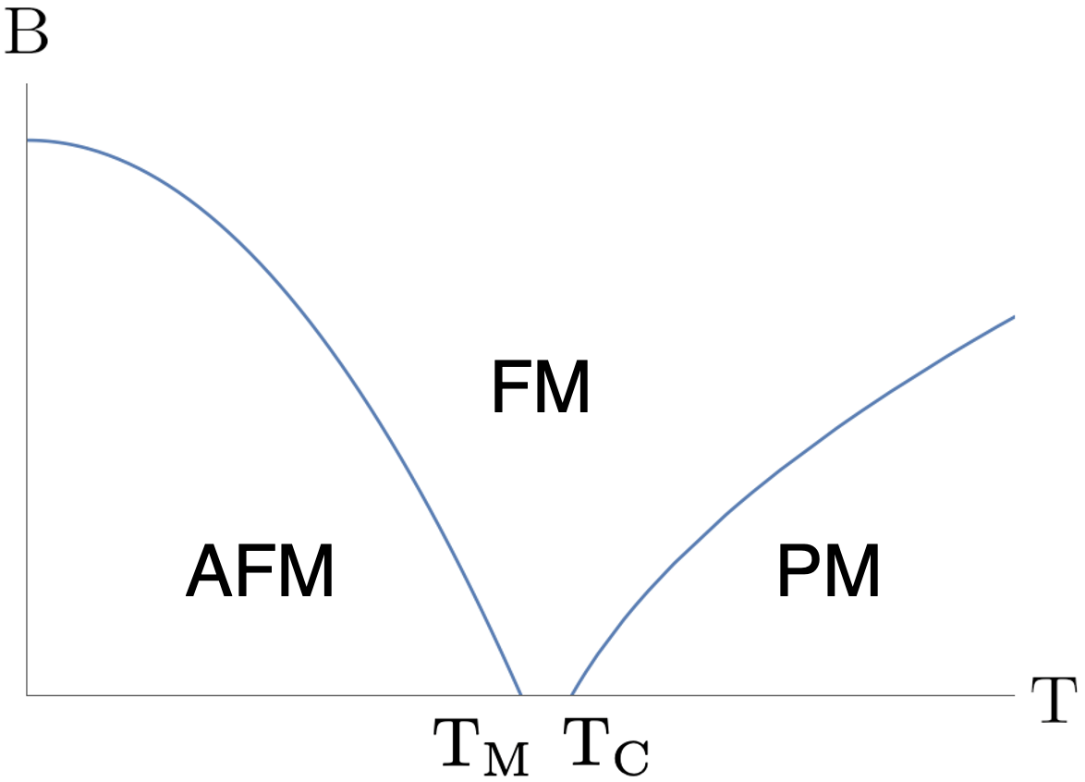


**Figure S9** **Phase diagram of Ni_2_Mn_1.4_In_0.6_**
